# Bioinspired Lipase Immobilized Membrane for Improving Hesperidin Lipophilization

**DOI:** 10.3390/antiox11101906

**Published:** 2022-09-26

**Authors:** Shanxiu Ming, Shuyi Li, Zhe Chen, Xujun Chen, Feifei Wang, Shaonan Deng, Krystian Marszałek, Zhenzhou Zhu, Wenxiang Zhang, Francisco J. Barba

**Affiliations:** 1 National R&D Center for Se-Rich Agricultural Products Processing Technology, Hubei Engineering Research Center for Deep Processing of Green Se-Rich Agricultural Products, School of Modern Industry for Selenium Science and Engineering, Wuhan Polytechnic University, Wuhan 430205, China; 2 Hubei Nanbai Shengtainongye Co., Ltd., Enshi 445000, China; 3 Prof. Wacław Dąbrowski Institute of Agricultural and Food Biotechnology—State Research Institute, Department of Fruit and Vegetable Product Technology, 36 Rakowiecka St., 02-532 Warsaw, Poland; 4 Department of Food Technology and Human Nutrition, Institute of Food Technology and Nutrition, College of Natural Science, University of Rzeszow, Zelwerowicza 2D, 35-601 Rzeszow, Poland; 5 Biological and Environmental Science and Engineering Division, Water Desalination and Reuse Research Center, King Abdullah University of Science and Technology, Riyadh 11543, Saudi Arabia; 6 Department of Preventive Medicine and Public Health, Food Science, Toxicology and Forensic Medicine, Faculty of Pharmacy, University of Valencia, 46100 Valencia, Spain

**Keywords:** *Candida antarctica* lipase B, enzymatic esterification, membrane separation, hesperidin lipophilization, bioinspired lipase immobilized membrane

## Abstract

Lipophilization is a promising way to improve the bioavailability of flavonoids. However, the traditional enzymatic esterification methods are time-consuming, and present low yields and purity. Herein, a novel membrane-based lipophilization technology—bioinspired lipase immobilized membranes (BLIMs), including CAL-B@PES, CAL-B@PDA/PES and GA/CAL-B@PDA/PES— were fabricated to improve the antioxidant flavanone glycoside hesperidin lipophilization. Via reverse filtration, PDA coating and GA crosslinking, *Candida antarctica* lipase B (CAL-B) was stably immobilized on membrane to fabricate BLIMs. Among the three BLIMs, GA/CAL-B@PDA/PES had the greatest enzyme activity and enzyme loading, the strongest tolerance of changes in external environmental conditions (temperatures, pH, heating time, storage time and numbers of cycles) and the highest hesperidin esterification efficiency. Moreover, the optimal operating condition for GA/CAL-B@PDA/PES fabrication was the CAL-B concentration of 0.36 mg/mL, operation pressure of 2 bar, GA concentration of 5% and crosslinking time of 1 h. Afterwards, the hesperidin esterification process did not affect the micromorphology of BLIM, but clearly improved the BLIM permeability and esterified product efficiency. The present study reveals the fabrication mechanism of BLIMs and offers insights into the optimizing strategy that governs the membrane-based lipophilization technology process.

## 1. Introduction

Flavonoids, a type of secondary metabolites of phenolic plants in nature, have a strong antioxidant value [1,2,3,4]. However, they are not easily absorbed into the body, due to the poor liposolubility, seriously limiting their applications [5]. As a type of flavonoid mainly found in sweet oranges and other citrus fruits, leaves and peels, hesperidin has the advantages of antioxidant, anticancer, antihypertension, antibacterial and vasodilator activities [6,7,8]. To promote the application value of this compound, enzymatic esterification has been widely utilized to improve its lipophilicity and bioavailability [9].

Lipase, with high selectivity and specificity, is regarded as the desirable choice for the acylation reaction [10,11]. Nevertheless, the free lipase cannot be recycled after the acylation reaction, making continuous production difficult, and resulting in a high operating cost [12,13,14]. To overcome the above stated disadvantages during the biocatalysis process, bioinspired lipase immobilized membranes (BLIMs), which immobilize lipase on the membrane surface or into the membrane pore, can combine catalysis and separation into one step, as well as to achieve lipase recycling, greatly improving the enzymatic esterification efficiency [15]. Additionally, BLIMs also clearly elevate the enzymatic activity and stability [16,17,18]. Guimarães et al. [19] found that *Candida antarctica* lipase B (CAL-B) immobilized on a PES membrane markedly strengthened the enzymatic activity and CAL-B reusability. Nevertheless, leakage and inactivation of CAL-B inevitably occurred during long-term operation because the CAL-B immobilization on the membrane surface through simple van der Waals force and hydrophobic interaction was not stable [20]. Thereby, it is necessary to improve the enzyme loading and enzymatic activity by membrane surface modification, for BLIMs fabrication.

In recent years, covalent binding and cross-linking have been developed to modify the membrane for better application performance [21,22,23]. For instance, polydopamine (PDA) can be synthesized by self-polymerization of dopamine (DA) under alkaline and oxygen conditions and has been widely utilized for membrane modification and enzyme immobilization via the introduction of active groups on the enzyme surface [24,25,26,27]. Touqeer et al. [28] found that PDA not only provided the active functional groups for the stable attachment of lipase, but also effectively enhanced its membrane anti-fouling ability with its greater hydrophilicity [29,30]. Glutaraldehyde (GA), as a relative cheap crosslinking agent, has also been frequently utilized for enzyme immobilization. The aldehyde group of GA makes it easier to quickly cross-link with amino groups on different enzyme molecules to form enzyme aggregates and increase the enzyme loading [31]. Neelam et al. [32] immobilized diamine oxidase molecules on a membrane surface to form a double-layer of immobilized enzyme via covalent bonding using GA to improve the enzyme loading on the membrane, the stability and reusability of the enzyme, and the adaptability to extreme environments.

Both PDA coating and GA co-deposition show a fine stability for effective enzyme loading. PDA, as a functional coating, has strong adhesion, and its rich active groups can covalently bind enzyme molecules to immobilize enzyme. However, under the conditions of external mechanical agitation and high temperature, the poor interfacial binding force between enzyme molecules leads to enzyme molecule leakage and enzyme activity decline [25]. GA, as a crosslinking agent, has abundant aldehyde groups to cross-link enzyme molecules to form an aggregate immobilized enzyme [26]. However, the crosslinking method has been rarely used alone, and is usually combined with other immobilized methods to achieve better immobilized effect [33]. In PDA coating and GA co-deposition, the GA addition effectively reduces the flow of enzyme molecules and increases the physical entanglement and chemical cross-linking between enzyme molecules and carrier, while maximally improving the stability of the immobilized enzyme and enlarging the enzyme loading [26]. PDA can better encapsulate the enzyme molecules in the membrane support layer, preventing the leakage of enzyme molecules in the washing process, and afterwards enhancing the interface binding force between the enzyme molecules and the carrier, so as to retain the enzyme activity [21]. Thus, the development of bioinspired lipase immobilized membrane (BLIM) with complete structure and optimized function, by suitable PDA coating and GA co-deposition, is expected to improve the enzymatic esterification of BLIMs.

In this work, PDA coating and GA co-deposition are utilized to immobilize CAL-B on the membrane surface to fabricate BLIMs with optimized structure and desirable performance. Then, the effect of fabrication conditions, including temperature, pH value, storage time, on the lipophilization ability, reusability, and stability of BLIMs, are clarified. Afterwards, the possible mechanisms of enzymatic esterification in BLIMs are discussed. The successful implementation of BLIM will provide an alternative method for the efficient production of flavonoid esterification and the improvement of its bioavailability.

## 2. Materials and Methods

### 2.1. Materials

A dead-end filtration cell (Amicon 8050, Millipore Corporation, Billerica, MA, USA) with a total volume of 50 mL and an effective area of 13.4 cm^2^ was used for the BLIM tests. PES ultrafiltration (UP030) with a molecular weight cut-off of 30 kDa was purchased from MICRODYN-NADIR. CAL-B (5000 LU/g) and Bradford Protein Concentration Determination Kit were bought from Beijing Cliscent Technology Co., Ltd., Beijing, China and Shanghai Biyuntian Biotechnology Co., Ltd., Shanghai, China respectively. N-heptane, sodium hydroxide, disodium hydrogen phosphate, and sodium dihydrogen phosphate were purchased from Sinopharm Group Co., Ltd. Lauric acid 99%, dopamine hydrochloride (DA⋅HCl), 2-methyl-2-butanol, and dimethyl sulfoxide (DMSO) were bought from Aladdin (Shanghai, China). Glutaraldehyde 50% and hesperidin 95% were purchased from Tianjin Damao Chemical Reagent Factory and Energy Chemical (Beijing, China), respectively. All chemicals were used as received without further purification.

### 2.2. Fabrication of Lipase Immobilized Membranes

**Figure 1:**
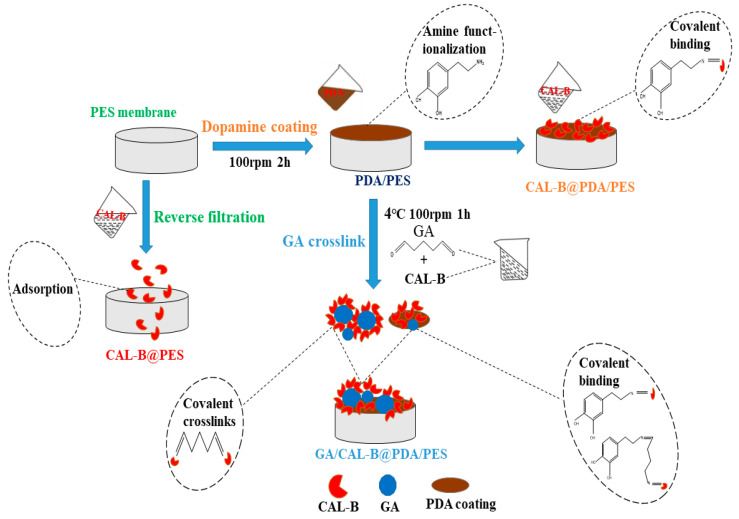
Schematic diagram for BLIMs’ fabrication.

The BPMs were fabricated via PDA coating followed by GA co-deposition, as shown in Figure 1. There were 3 forms of BLIM: CAL-B@PES, CAL-B@PDA/PES and GA/CAL-B@PDA/PES. Their fabrication processes are described as follows (Figure 1).

CAL-B@PES: Firstly, a new membrane was immersed in 50% ethanol for 2 min to remove some non-binding molecules and transferred to deionized water for storage overnight. Then, the obtained membrane was placed at the bottom of the filtration cell, and 10 mL of CAL-B solution (0.36–2.16 mg/mL, prepared with a phosphate buffer of pH = 7) was added for enzyme immobilization under a transmembrane pressure (TMP) of 1–4 bar. The agitation speed was fixed at 100 rpm. Finally, 10 mL of phosphate buffered solution (PBS, pH = 7) was used to wash the non-immobilized enzyme at 2 bar to obtain the prepared CAL-B@PES.

CAL-B@PDA/PES: The 50% ethanol-treated new membrane as mentioned above was placed at the bottom of filtration cell, and 10 mL of 2 g/L PDA solution (prepared with tris HCl buffer, pH = 8.5) was added. The mixture was slowly stirred for 1–5 h to ensure the PDA coated the membrane. To avoid membrane pore blocking, 30 mL PBS (pH = 7) buffer solution was utilized to clean the dopamine without polymerization at 2 bar to obtain the PDA/PES membrane. After that, CAL-B immobilization was carried out by repeating the operations stated for CAL-B@PES fabrication, to form CAL-B@PDA/PES.

GA/CAL-B@PDA/PES: 10 mL of 2.5–12.5% GA solution and 10 mL of CAL-B solution (0.18–1.08 mg/mL, prepared with a phosphate buffer, pH = 7) were mixed and reacted at 4 °C and 100 rpm for 0–2 h. After the reaction, all the GA/CAL-B solution was added into the filtration cell of the above modified PDA/PES membrane for CAL-B immobilization at 1–4 bar and 100 rpm agitation. Subsequently, 10 mL of PBS buffer solution (pH = 7) was used to wash the unstable enzyme at 2 bar, to obtain the GA/CAL-B@PDA/PES.

Finally, these BLIMs were soaked in 10 mL of PBS buffer (pH = 7) and stored overnight at 4 °C. The permeate solution in BLIMs’ fabrication, and the preservation solution, were accumulated in order to calculate the amount of immobilized enzyme by mass balance.

### 2.3. Characterization and Analysis

The micromorphology of the BLIMs were evaluated via scanning electron microscope (SEM, S4800, Hitachi Ltd., Tokyo, Japan), while the component was measured by attenuated total reflection flourier transformed infrared spectroscopy (ATR-FTIR, Spectrum 100, Perkin Elmer, Inc., Waltham, MA, USA). X-ray photoelectron spectroscopy (XPS, ESCALAB250XI, Thermo Scientific, Waltham, MA, USA) was applied to analyze the chemical compositions of the membrane surface. The formation of acylated compounds was monitored by HPLC (Agilent 1100) equipped with a diode array detector on an Alltima C18 column (5 μm, 4.6 mm × 250 mm). The permeability of the BLIMs was measured by balance weighing. The ion mass of hesperidin laurate was accurately measured by high-resolution mass spectrometry (HRMS) (UltrafleXtreme MALDI TOF/TOF, Bruker Daltonics Co., Ltd., Billerica, MA, USA).

### 2.4. Measurement of CAL-B Esterification Activity

A reaction solution (20 mL) containing 0.6 mol/L lauric acid (10 mL) and 0.72 mol/L absolute ethanol (10 mL) with n-heptane as solvent was prepared in a 100 mL beaker. The BLIMs (or free enzyme) were placed into the beaker and incubated at 40 °C for 30 min in a shaking water bath at 200 rpm. Then 10 mL of 95% ethanol solution was used to terminate the reaction, followed by titration with 0.2 mol/L NaOH, using 0.1% phenolphthalein as an indicator. The esterification activity was defined as 1 µmol lauric acid consumed in the esterification reaction per min per mg enzyme (µmol mg^−1^ min ^−1^) [34].

The enzyme activity (µmol mg^−1^ min^−1^) was calculated using Equation (1):

(1) $$\mathrm{Enzyme}\;\mathrm{activity}\;\left(X\right)=\frac{V\times M\times 10^{3}}{E\times T}$$

where V is the difference between the volume of NaOH consumed by titrating the blank control and the sample. T, M and E are the reaction time (min), NaOH molarity (mol/L) and enzyme mass (mg), respectively.

The stability of free and immobilized enzymes was determined by Equation (2):

(2) $$\mathrm{Relative}\;\mathrm{activity}\;\left(\%\right)=\frac{R_{1}}{R_{t}}\times 100$$

where R_1_ is the enzyme activity under different conditions, and Rt is the highest enzyme activity.

The amount of immobilized enzyme was identified from the mass balance equation:

(3) $$M=M_{1}-C_{2}\times V_{2}-C_{3}\times V_{3}$$

where M_1_ is the total enzyme amount (mg) used for BLIM fabrication, while *C_2_* and *C_3_* are lipase concentration (mg/mL) in permeate solution and retentate solution, respectively. V_2_ and V_3_ are the volume (mL) of permeate solution and retentate solution, respectively.

The permeate flux (J) was calculated using Equation (4) [35]:

(4) $$J=\frac{V}{S\times T}$$

where *V* is the volume of permeate (L), *T* is the filtration time (h), and *S* is the effective membrane area (m^2^).

The permeability (L) was calculated using Equation (5):

(5) $$L=\frac{J}{\mathrm{TMP}}$$

where TMP represents the transmembrane pressure (bar).

### 2.5. Stability Test of BLIMs

The effect of temperature, pH, heating time, storage time and numbers of cycles, on the relative activity, was studied. The BLIMs were incubated at 30–80 °C for 30 min to determine the activity of the immobilized enzyme, indicating the temperature tolerance. For pH tolerance measurement, the BLIMs were incubated in different buffer solutions (pH = 5–9) for 2.0 h at room temperature, and then the enzyme relative activity was tested. To study the thermal stability, the BLIMs were incubated in a water bath at 50 °C for 5–30 min followed by the enzyme activity measurement. The storage stability of the BLIMs was tested after 18 days of storage at 4 °C; the enzyme activity was determined every three days. The re-usability of the BLIMs was identified by the enzyme activity after multiple applications in the ethanol-lauric acid system, as stated in Section 2.4.

### 2.6. Enzymatic Esterification of Hesperidin with BLIMs

Firstly, the hesperidin and lauric acid were vacuum-dried at 30 °C for more than 24 h. Then hesperidin and lauric acid were dissolved in 2-methyl-2-butanol at a molar ratio of 1:5. A small amount of DMSO was added to dissolve the hesperidin so that the total volume of the reactional mixture was 20 mL. The prepared reaction mixture was placed in a water bath of 50 °C for 15 min for complete dissolution, and then poured into the filtration cell fixed by the above-mentioned CAL-B@PES, CAL-B@PDA/PES and GA/CAL-B@PDA/PES membranes, with a temperature of 60–70 °C, TMP of 1–2 bar and agitation speed of 100 rpm, for enzymatic esterification reaction. After accumulating the permeate, 2-methyl-2-butanol was removed by rotary evaporation, and a yellow solid was obtained by freeze drying. Finally, the obtained samples were dissolved in chromatographic methanol and filtered through a 0.22 μm microporous membrane. The unreacted hesperidin content was determined by HPLC to calculate the lipophilization rate [19,36].

### 2.7. Data Analysis

All tests were repeated at least 3 times. The errors were controlled below 5% and average values were calculated and demonstrated in the Figures and Tables.

## 3. Results

### 3.1. The Characterization of BLIMs

**Figure 2:**
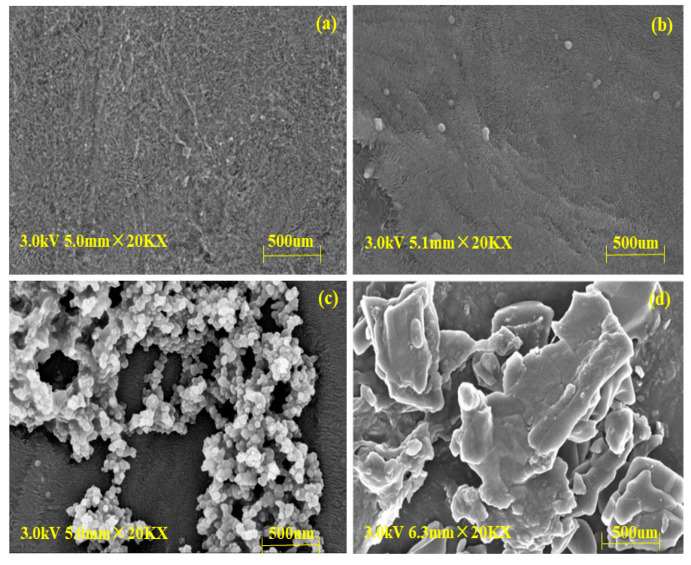
SEM images of BLIMs: (**a**) PES membrane, (**b**) CAL-B@PES, (**c**) CAL-B@PDA/PES, and (**d**) GA/CAL-B@PDA/PES.

The SEM images of (a) new membranes, (b) CAL-B@PES, (c) CAL-B@PDA/PES, and (d) GA/CAL-B@PDA/PES are displayed in Figure 2. Compared with the new membrane, Figure 2b–d shows fibers with sporadic globules of immobilized CAL-B on the membrane support layer, indicating the stable immobilization of CAL-B on the new membrane. Moreover, Figure 2d illustrates more and larger sporadic globules of immobilized CAL-B than Figure 2c, on account of the aggregation of GA crosslinked CAL-B, increasing the enzyme loading on the membrane.

**Figure 3:**
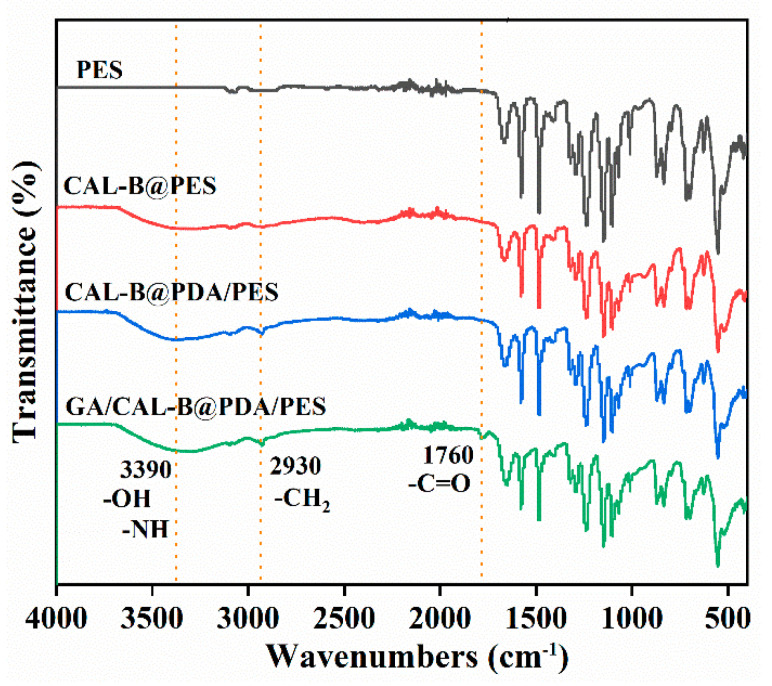
FTIR spectrum of BLIMs.

The functional group changes of the new membrane, CAL-B@PES, CAL-B@PDA/PES and GA/CAL-B@PDA/PES were analyzed by FTIR spectrum (Figure 3). In comparison with the new membrane, CAL-B@PES had a new peak value at 3390 cm^−1^, which belonged to the stretching vibration of -NH and -OH groups, confirming the successful immobilization of CAL-B on the membrane [13]. Furthermore, compared with CAL-B@PES, the peak stretching vibration of PDA-coated CAL-B@PDA/PES enhanced at 3390 cm^−1^, and some new bands appeared at the range of -CH_2_ (2930 cm^−1^), which provided evidence for the successful PDA coating on the new membrane [37]. The characteristic peak of carbonyl group appeared in GA/CAL-B@PDA/PES at 1760 cm^−1^, implying that GA was successfully adhered to PDA/PES membrane [33].

Table 1 shows the element composition for the different BLIMs. Compared with the new membrane, the concentrations of sulfur and nitrogen on the surface of CAL-B@PES increased to 0.44% and 5.12%, respectively, implying that CAL-B successfully immobilized on the membrane. Similarly, the appearance of nitrogen element on the surface of PDA/PES membrane was due to the presence of oxygen element and nitrogen element in PDA itself, which also confirmed the stable PDA coating on the new membrane [38]. Additionally, after being coated by PDA, the higher concentrations of oxygen and nitrogen for CAL-B@PDA/PES and GA/CAL-B@PDA/PES were derived from the successful immobilization of CAL-B, thus, PDA coating and GA crosslinking can be utilized for BLIM fabrication.

**Figure 4:**
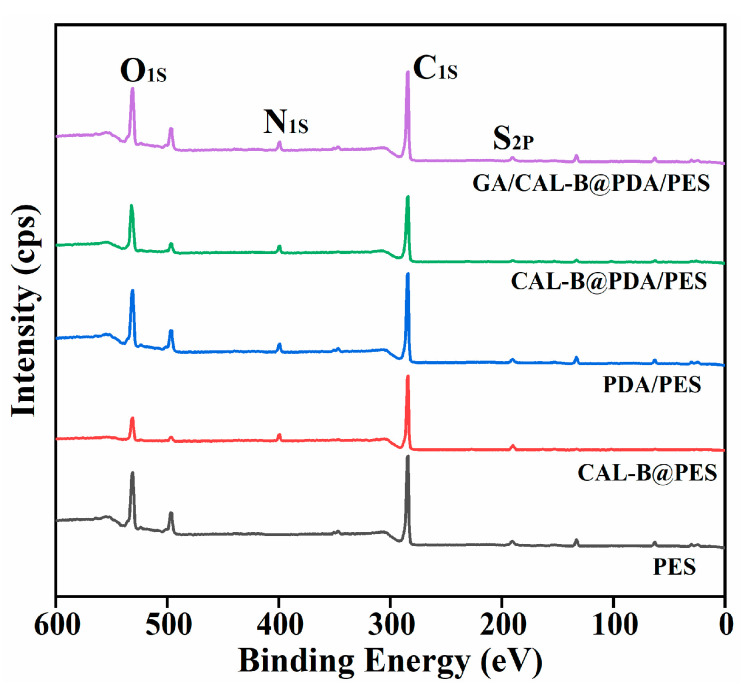
XPS spectrum of the BLIMs.

Figure 4 illustrates the XPS spectrum of BLIMs. The new membrane presented S_2P_ spectrum at 165.78 eV, C_1s_ spectrum at 284.48 eV and O_1s_ spectrum at 531.38 eV. Compared with the new membrane, N_1s_ spectrum fluctuation was observed at 399.28 eV for PDA/PES membrane, confirming the successful PDA coating on the membrane. Moreover, for the three kinds of BLIMs, it was found that S_2P_ spectrum, O_1s_ spectrum and N1s spectrum exhibited different degrees of stretching vibration, on account of the CAL-B adsorption on the membrane. Thereby, the BLIMs possessed the best CAL-B immobilization effect.

### 3.2. The BLIMs Fabrication

#### 3.2.1. Effect of Fabrication Operating Condition on CAL-B@PES Performance

**Figure 5:**
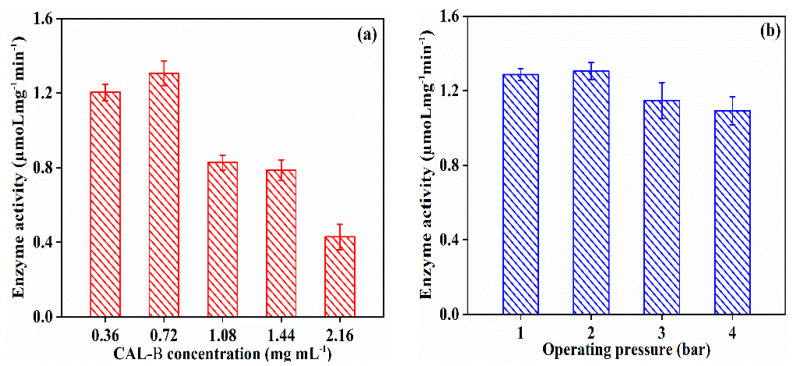
Enzyme activity of CAL-B@PES at different fabrication operating conditions: (**a**) CAL-B concentration, and (**b**) operating pressure.

As illustrated in Figure 5, with the elevation of CAL-B concentration, the activity of immobilized CAL-B grew slowly, and then decreased. This might be ascribed to the CAL-B saturation on membrane at CAL-B concentration of 0.72 mg/mL, while the higher enzyme concentration led to membrane pore blocking, which covered the enzyme active sites, resulting in the decrease in enzyme activity. The enzyme activity maintained at 1.306 µmoL mg^−1^ min^−1^ when the operating pressure increased to 2 bar, then decreased to 1.093 µmoLmg^−1^ min ^−1^ at 4 bar, because of the leakage and denaturation of CAL-B under the higher pressure. Thus, the CAL-B concentration of 0.72 mg/mL and operating pressure of 2 bar were the optimal operating conditions for CAL-B@PES fabrication.

#### 3.2.2. Effect of Fabrication Operating Condition on CAL-B@PDA/PES Performance

**Figure 6:**
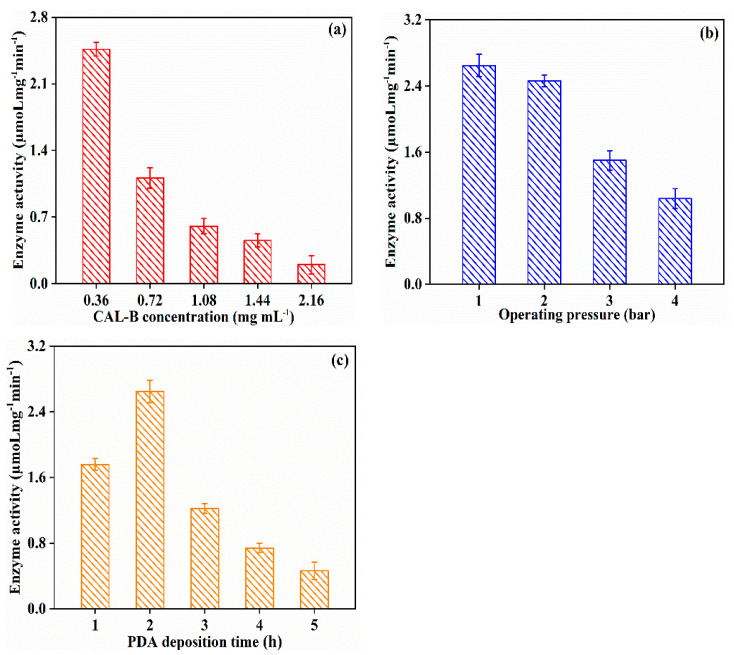
Enzyme activity of CAL-B@PDA/PES at different fabrication operating conditions: (**a**) CAL-B concentration, (**b**) operating pressure, and (**c**) PDA deposition time.

Different from CAL-B@PES, the increase in CAL-B concentration (0.36 → 2.16 mg/mL) resulted in a gradual declination of enzyme activity (2.463 → 0.201 µmoL mg^−1^ min^−1^) for CAL-B@PDA/PES (shown in Figure 6a). The ionization of the active site of CAL-B in alkaline condition during the PDA coating process led to the same negative charge of both lipase and PDA/PES membrane and the change of lipase conformation, then decrease of the enzyme activity [39]. Figure 6b presents the effect of fabrication operating pressure on the enzyme activity. The enzyme activity of immobilized CAL-B reached the highest value at operating pressure of 1 bar. In Figure 6c, the maximum enzyme activity was 2.648 µmoL mg^−1^ min^−1^ at a PDA coating time of 2 h, while the longer PDA coating time caused a lower enzyme activity, because the generation of more negative charges on PDA/PES membrane was able to reversibly inhibit CAL-B and reduce the enzyme activity. Therefore, the enzyme activity of CAL-B@PDA/PES reached the optimal value at the CAL-B concentration of 0.36 mg/mL, operating pressure of 1 bar, and PDA coating time of 2 h.

#### 3.2.3. Effect of Fabrication Operating Condition on GA/CAL-B@PDA/PES Performance

**Figure 7:**
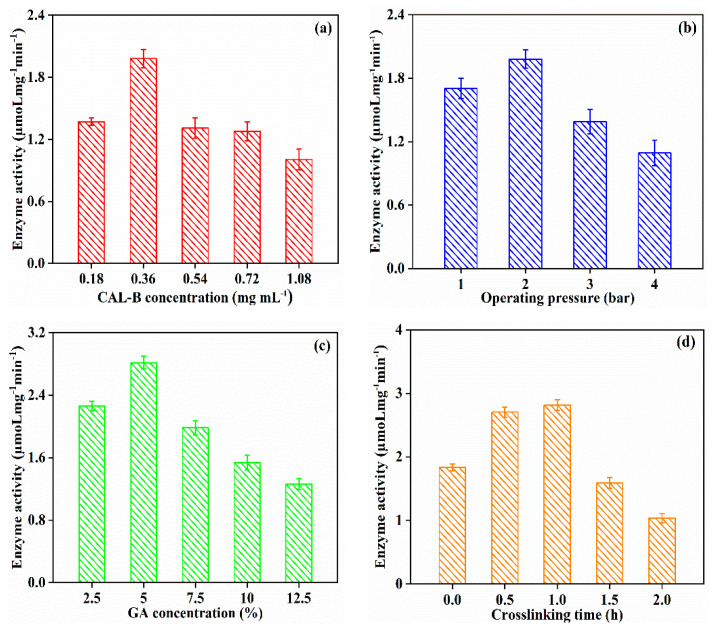
Enzyme activity of GA/CAL-B@PDA/PES at different fabrication operating conditions: (**a**) CAL-B concentration, (**b**) operating pressure, (**c**) GA concentration, and (**d**) crosslinking time.

Figure 7 indicates that the enzyme activity increased to 2.815 µmoL mg^−1^ min^−1^, when GA concentration increased from 2.5% to 5%, on account of the better cross-linking between the enzyme molecules. However, further augmentation of GA concentration brought about more Schiff base reactions with the amino group on the enzyme molecule, causing denaturation of the enzyme active site, thereafter diminishing the enzyme activity [40]. Due to better immobilization of CAL-B, the longer crosslinking time (<1 h) obtained a greater enzyme activity. However, the prolonged crosslinking time (>1 h) tremendously reduced the enzyme activity. Excessive crosslinking may be related to the CAL-B covering on the membranes, decreasing the activity of GA/CAL-B@PDA/PES. Thus, the optimal operating condition for GA/CAL-B@PDA/PES fabrication was a CAL-B concentration of 0.36 mg/mL, operation pressure of 2 bar, GA concentration of 5% and crosslinking time of 1 h.

### 3.3. The Enzyme Activity and Enzyme Loading for Free and Immobilized CAL-B

**Figure 8:**
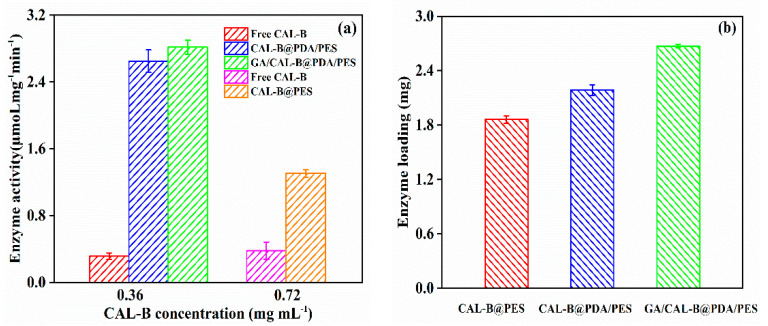
(**a**) Enzyme activity, and (**b**) enzyme loading, for BLIMs.

Figure 8a depicts the enzyme activity of free CAL-B and the three BLIMs. In most cases, the enzyme activity decreased after immobilization, because of the structural change of the enzyme, resulting in the modification of catalytic amino acid residues [41]. Nevertheless, the enzyme activity significantly improved after immobilization, thus, the enzyme activity center became a hydrophobic pocket inside the enzyme molecule. After enzyme immobilization on the hydrophilic PDA layer or membrane support layer, the membrane surface became hydrophobic and the substrate was more likely to react with the enzyme in the hydrophobic environment. Moreover, GA/CAL-B@PDA/PES had the highest enzyme activity among the fabricated BLIMs, because its immobilization method could better protect the enzyme molecules and improve the enzyme stability [42].

Figure 8b depicts the enzyme loading for the different BLIMs. Due to the additional active site (phenol hydroxyl, provided by the PDA coating layer), which could react with amino group of CAL-B to form a covalent binding [43], CAL-B@PDA/PES possessed the better enzyme loading than that of CAL-B@PES, which only adsorbed the enzyme through van der Waals force and hydrophobic interaction. Furthermore, the enzyme loading of GA/CAL-B@PDA/PES was even higher than that of CAL-B@PDA/PES. With the active functional groups (aldehyde group), GA was grafted onto the PDA coating by Schiff base reaction to furtherly modify the membrane surface, improving the CAL-B immobilization efficiency via crosslinking [32]. Thereby, among the three BLIMs, GA/CAL-B@PDA/PES had better enzyme activity and enzyme loading capacity.

### 3.4. Stability Analysis of BLIMs

**Figure 9:**
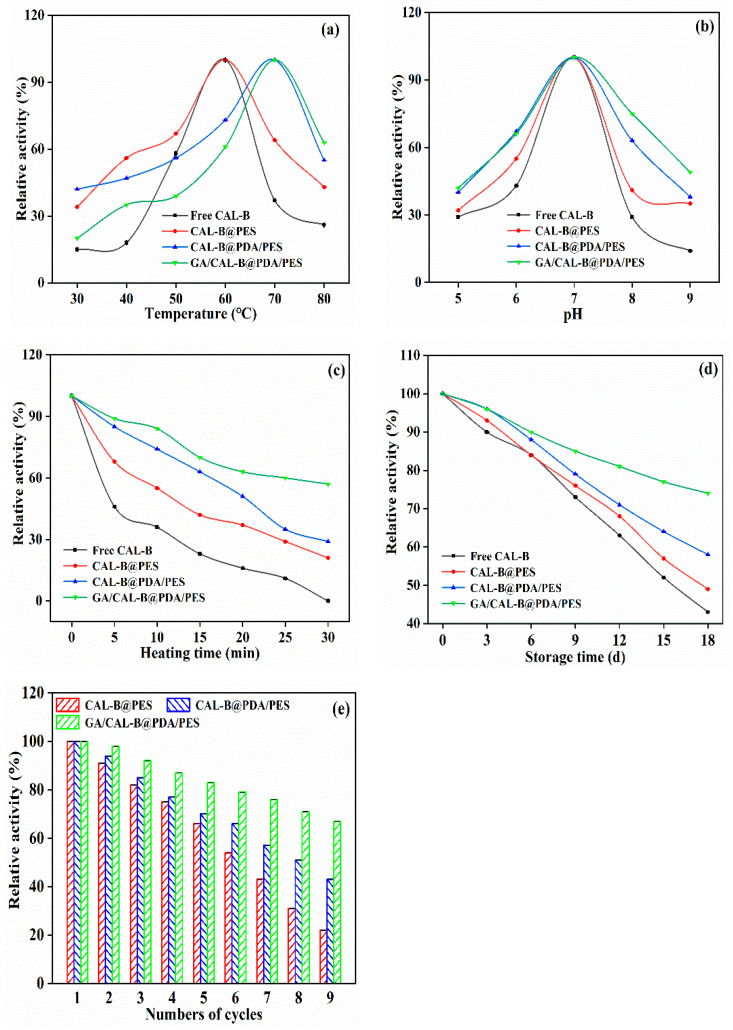
Relative activity at various temperatures (**a**), pH (**b**), heating time (**c**), storage time (**d**) and numbers of cycles (**e**).

Figure 9 shows the BLIMs’ relative activity at various temperatures, pH, heating time, storage time and numbers of cycles. As shown in Figure 9a, the highest relative activity of free CAL-B and CAL-B@PES was 100% at 60 °C, whereas they decreased to 26% and 43% at 80 °C, respectively. At the same time, CAL-B@PDA/PES and GA/CAL-B@PDA/PES followed the same trend. They possessed a relative activity of 100% at 70 °C, then the relative activity reduced to 55% and 63% at 80 °C, which were 1.3 and 1.5 times (only 43%) of CAL-B@PES, respectively. The higher relative activity of GA/CAL-B@PDA/PES at 80 °C may have been due to the buffering effect of the aldehyde group and amino group abundant in GA and PDA, which made the enzyme microenvironment temperature lower than that of the original solution [44].

Figure 9b illustrates the impact of pH on the relative activity. At the same pH value, the freer CAL-B was more greatly inactivated than immobilized CAL-B, which may have been due to the excessive dissociation of active sites in the free CAL-B under strong acid and alkali conditions, decreasing the enzyme activity [43]. When the pH value increased to 7.0 or even higher, GA/CAL-B@PDA/PES had higher enzyme activity than CAL-B@PDA/PES and CAL-B@PES, on account of the conformation change of enzyme molecules caused by the interaction between enzyme alkaline residues and glutaraldehyde during the crosslinking process, thus, reducing the fluidity of the internal molecules and enhancing the enzyme tolerance to pH [45]. Additionally, the hydroxyl groups on PDA/PES membrane also prevented the excessive dissociation and inactivation of enzyme active groups, to a certain extent [46].

As expected, the thermal stability of the BLIMs was significantly elevated compared with the free CAL-B (displayed in Figure 9c). At 50 °C for 30 min, the relative activity was 21%, 29%, and 57%, for CAL-B@PES, CAL-B@PDA/PES and GA/CAL-B@PDA/PES, respectively. The free CAL-B was basically inactivated, which was heated to break the bond, causing protein denaturation and enzyme molecule inactivation. However, GA/CAL-B@PDA/PES of GA crosslinking enzyme molecule had a protective effect on the enzyme protein, due to the active group on the PDA/PES membrane. Moreover, GA, as a cross-linking agent, made enzyme molecules intertwine together to form a protective layer, which also played a protective role on enzyme molecules. The double layer protection further reduced the sensitivity of the enzyme structure to temperature and improved the thermal stability of GA/CAL-B@PDA/PES.

According to Figure 9d, the relative activity for free and immobilized CAL-B gradually decreased with time. During the storage time of 18 days at 4 °C, the relative activities for free CAL-B and CAL-B@PES were both only 40%. The relative activities of CAL-B@PDA/PES and GA/CAL-B@PDA/PES remained at 58% and 74%, respectively, since the enzyme immobilization significantly improved the storage stability of CAL-B.

In order to evaluate the reusability, nine cycles of repeated experiments were conducted. As shown in Figure 9e, the relative activity of CAL-B@PES decreased by 78% after the ninth cycle, because some weak binding enzyme was lost during the long-term operation [47]. The residual activity of CAL-B@PDA/PES decreased by about 60%, because of the removal of immobilized free CAL-B from PDA/PES, mechanical damage in the reaction process, and the erosion of weak CAL-B during the washing process [10]. For GA/CAL-B@PDA/PES, the relative activity remained about 70%, indicating that it exhibited its desirable application prospect. Thence, GA/CAL-B@PDA/PES had the strongest tolerance of the enzyme structure to the external environment.

### 3.5. Hesperidin Lipophilization by BLIMs

**Figure 10:**
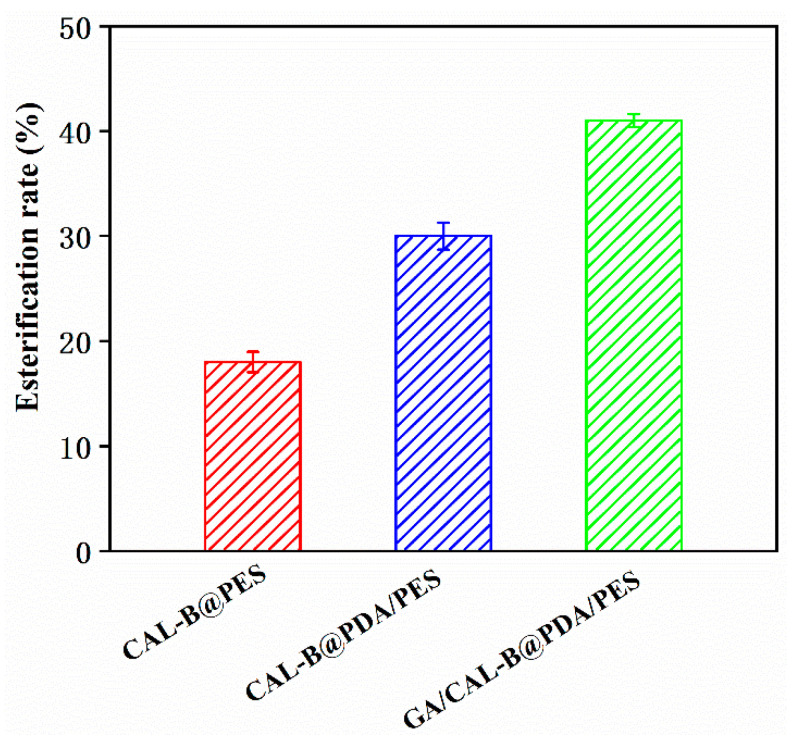
Esterification rate of hesperidin for different BLIMs.

The hesperidin esterification rates of BLIMs are presented in Figure 10. The hesperidin esterification rate of GA/CAL-B@PDA/PES reached 40.9% after 32 min, which exceeded CAL-B@PES and CAL-B@PDA/PES. The new PES membrane was modified by PDA coating and GA crosslinking to form the structure of a biomimetic carrier. The introduction of GA enabled enzyme molecules to become enzyme aggregates through covalent cross-linking, for better encapsulating enzyme molecules in the PDA/PES membrane support layer. Additionally, the active group (phenolic hydroxyl) in the enzyme molecule further reacted with the active group (amino) in the PDA coating to achieve the immobilization of the enzyme again, enhancing the interaction between the membrane and the enzyme, thus, the stability of GA/CAL-B@PDA/PES also improved. Thus, the fabrication method of GA/CAL-B@PDA/PES enhanced the hesperidin esterification efficiency [10].

### 3.6. BLIMs Performance

#### 3.6.1. Morphologies of BLIMs after Esterification

**Figure 11:**
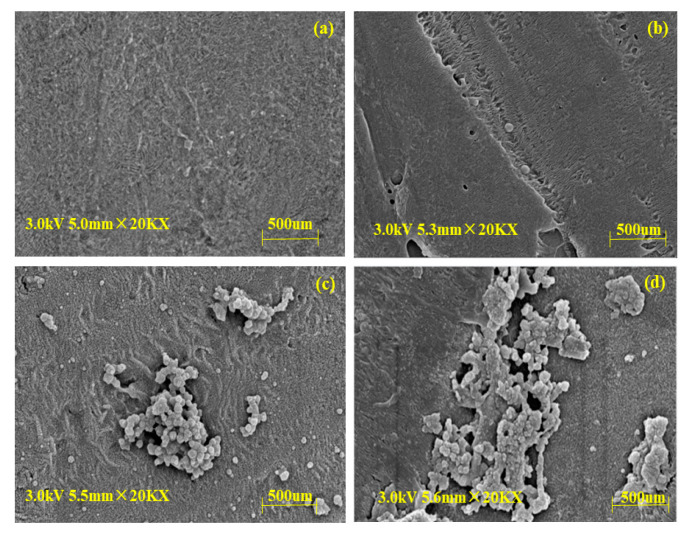
SEM images of BLIMs: (**a**) PES membrane, (**b**) CAL-B@PES, (**c**) CAL-B@PDA/PES, and (**d**) GA/CAL-B@PDA/PES, after esterification.

The morphologies of the BLIMs after hesperidin esterification were observed via SEM in Figure 11. Compared with the new membrane, Figure 11b–d shows that the membrane support layer became rougher and with larger porosity, since the thermal expansion during hesperidin esterification process may enlarge membrane pores [48]. Among them, the GA/CAL-B@PDA/PES had the smallest change in membrane pore, because the dopamine and CAL-B covering the GA/CAL-B@PDA/PES support layer reduced the surface porosity of the membrane, to a certain extent. During the enzymatic esterification of hesperidin by GA/CAL-B@PDA/PES at high temperature, the temperature did not cause serious damage to membrane material, thus, GA/CAL-B@PDA/PES had excellent application performance.

#### 3.6.2. BLIMs Permeability

**Figure 12:**
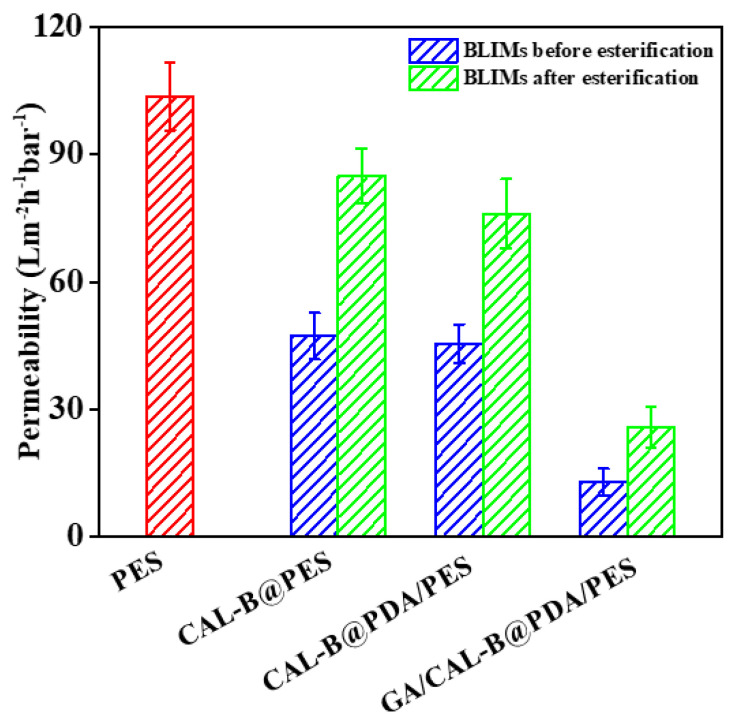
Permeability of BLIMs before and after esterification.

Figure 12 illustrates the BLIMs permeability before and after esterification. The permeability of new PES membrane, CAL-B@PES, CAL-B@PDA/PES and GA/CAL-B@PDA/PES were 103.6, 47.3, 45.51 and 13 L m^−2^ h^−1^ bar^−1^, respectively. Both PDA coating and GA co-deposition greatly enhanced the filtration resistance. Nevertheless, after hesperidin lipophilization, the permeability significantly increased to 84.9, 76.1 and 25.8 L m^−2^ h^−1^ bar^−1^, respectively, on account of the enlargement of membrane pore under the high temperature operation. This was consistent with the above results in Section 3.6.2. Thence, the esterification process is benefit for boosting the operation efficiency of BLIMs.

#### 3.6.3. The Esterified Products

**Figure 13:**
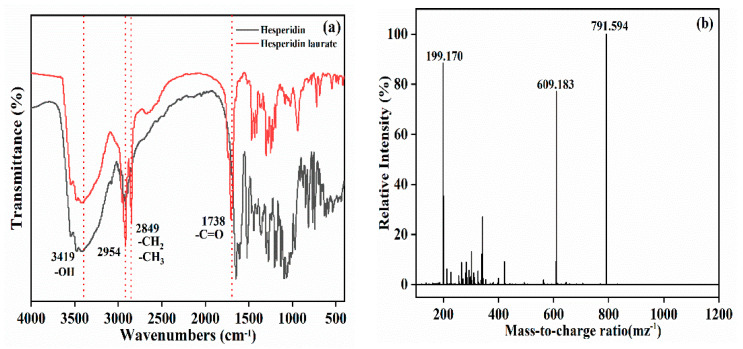
(**a**) FTIR spectra of hesperidin and hesperidin laurate, and (**b**) HRMS of hesperidin laurate.

Figure 13a displays the FTIR spectra for hesperidin and hesperidin laurate. Compared with hesperidin, -OH stretching vibration of hesperidin laurate at 3419 cm^−1^ decreased, indicating the formation of C = O by -OH acylation. While the antisymmetric and symmetric stretching vibration absorption peak intensity of methyl and methylene at 2954–2849 cm^−1^ obviously increased. Additionally, the appearance of a new peak appeared at 1738 cm^−1^, which belonged to the characteristic absorption peak of the C = O group, also confirmed the production of hesperidin laurate [49]. Figure 13b shows the HRMS of hesperidin laurate. There were ion peaks at m/z of 199.170 and 609.183, implying the existence of lauric acid and hesperidin. Moreover, the ion peaks at 791.594 was consistent with the molecular weight of hesperidin laurate monoester [9]. Thereby, BLIMs were able to effectively improve the hesperidin lipophilization.

## 4. Conclusions

The present study revealed the fabrication mechanism and operating condition optimization of BLIMs, and investigated the membrane-based hesperidin lipophilization efficiency. The following conclusions can be made:

* Via reverse filtration, PDA coating and GA crosslinking, CAL-B could be stably immobilized on a membrane to fabricate BLIMs;

* The optimal operating condition for GA/CAL-B@PDA/PES fabrication was a CAL-B concentration of 0.36 mg/mL, operation pressure of 2 bar, GA concentration of 5% and crosslinking time of 1 h;

* Among the three BLIMs, GA/CAL-B@PDA/PES had the greatest enzyme activity and enzyme loading, the strongest tolerance of changes in external environmental conditions (temperatures, pH, heating time, storage time and numbers of cycles) and the highest hesperidin esterification efficiency;

* The hesperidin esterification process did not affect the micromorphology of the BLIM, but clearly improved the BLIM permeability and esterified product efficiency.

The information presented in this study will undoubtedly benefit research on membrane-based hesperidin lipophilization.

## Figures and Tables

**Table 1:** Elements analysis on the new membrane, CAL-B@PES, PDA/PES, CALB@PDA/PES and GA/CAL-B@PDA/PES.

Elements (%)	PES	CAL-B@ PES	PDA/PES	CAL-B@PD A/PES	GA/CAL-B@ PDA/PES
C	95.71	86.13	87.07	74.91	77.14
N	0	5.12	3.36	5.51	4.85
O	4.2	8.3	9.33	19.27	17.75
S	0.8	0.44	0.23	0.32	0.25

## Data Availability

The data presented in this study are available in article.

## References

[1] Lei D., Li J., Zhang C., Li S., Zhu Z., Wang F., Deng Q., Grimi N. (2022). Complexation of soybean protein isolate with β-glucan and myricetin: Different affinity on 7S and 11S globulin by QCM-D and molecular simulation analysis. Food Chem. X.

[2] Li S., Xu H., Sui Y., Mei X., Shi J., Cai S., Xiong T., Carrillo C., Castagnini J.M., Zhu Z. (2022). Comparing the LC-MS phenolic acids profiles of seven different varieties of brown rice (*Oryza sativa* L.). Foods.

[3] Guo Z., Huang Y., Huang J., Li S., Zhu Z., Deng Q., Cheng S. (2022). Formation of protein-anthocyanin complex induced by grape skin extracts interacting with wheat gliadins: Multi-spectroscopy and molecular docking analysis. Food Chem..

[4] Gao W., Zhang N., Li S., Li S., Zhu S., Cong X., Cheng S., Barba F.J., Zhu Z. (2022). Polysaccharides in selenium-enriched tea: Extraction performance under innovative technologies and antioxidant activities. Foods.

[5] Zhang M., Zhu S., Yang W., Huang Q., Ho C.-T. (2021). The biological fate and bioefficacy of citrus flavonoids: Bioavailability, biotransformation, and delivery systems. Food Funct..

[6] Cao R., Zhao Y., Zhou Z., Zhao X. (2017). Enhancement of the water solubility and antioxidant activity of hesperidin by chitooligosaccharide. J. Sci. Food Agric..

[7] Lee S., Kim H.J. (2022). Antioxidant activities of premature and mature mandarin (*Citrus unshiu*) peel and juice extracts. Food Sci. Biotechnol..

[8] Loizzo M.R., Sicari V., Tundis R., Leporini M., Falco T., Calabrò V. (2019). The influence of ultrafiltration of *Citrus limon* L. burm. cv femminello comune juice on its chemical composition and antioxidant and hypoglycemic properties. Antioxidants.

[9] Araújo M.E.M.B., Contesini F.J., Franco Y.E.M., Sawaya A.C.H.F., Alberto T.G., Dalfré N., Carvalho P.D.O. (2011). Optimized enzymatic synthesis of hesperidin fatty acid esters in a two-phase system containing lonic liquid. Molecules.

[10] Sun T., Dong Z., Wang J., Huang F., Zheng M. (2020). Ultrasound-assisted interfacial immobilization of lipase on hollow mesoporous silica spheres in a pickering emulsion system: A hyperactive and sustainable biocatalyst. ACS Sustain. Chem. Eng..

[11] Jafarian F., Bordbar A.-K., Razmjou A., Zare A. (2020). The fabrication of a high performance enzymatic hybrid membrane reactor (EHMR) containing immobilized candida rugosa lipase (CRL) onto graphene oxide nanosheets-blended polyethersulfone membrane. J. Membr. Sci..

[12] Thangaraj B., Solomon P.R. (2019). Immobilization of lipases—A review. Part I: Enzyme immobilization. Chembioeng. Rev..

[13] Widhyahrini K., Handayani N., Wahyuningrum D., Nurbaiti S., Radiman C.L. (2017). The microwave-assisted synthesis of polyethersulfone (PES) as a matrix in immobilization of candida antarctica lipase B (Cal-B). Bull. Chem. React. Eng..

[14] T.sriwong K., Matsuda T. (2022). Recent advances in enzyme immobilization utilizing nanotechnology for biocatalysis. Org. Process Res. Dev..

[15] Luo J., Song S., Zhang H., Zhang H., Zhang J., Wan Y. (2020). Biocatalytic membrane: Go far beyond enzyme immobilization. Eng. Life Sci..

[16] Cao T., Pázmándi M., Galambos I., Kovács Z. (2020). Continuous production of galacto-oligosaccharides by an enzyme membrane reactor utilizing free enzymes. Membranes.

[17] Sun H., Tang B., Wu P. (2017). Development of hybrid ultrafiltration membranes with improved water separation properties using modified superhydrophilic metal-organic framework nanoparticles. ACS Appl. Mater. Interfaces.

[18] Fan R., Burghardt J.P., Prell F., Zorn H., Czermak P. (2020). Production and purification of fructo-oligosaccharides using an enzyme membrane bioreactor and subsequent fermentation with probiotic bacillus coagulans. Sep. Purif. Technol..

[19] Guimarães M., Pérez-Gregorio M., Mateus N., Freitas V.d., Galinha C.F., Crespo J.G., Portugal C.A.M., Cruz L. (2019). An efficient method for anthocyanins lipophilization based on enzyme retention in membrane systems. Food Chem..

[20] Thuanthong M., Gobba C.D., Sirinupong N., Youravong W., Otte J. (2017). Purification and characterization of angiotensin-converting enzyme inhibitory peptides from nile tilapia (*Oreochromis niloticus*) skin gelatine produced by an enzymatic membrane reactor. J. Funct. Foods.

[21] Chen Z., Sun Z., Ming S., Li S., Zhu Z., Zhang W. (2020). Bioinspired proteolytic membrane (BPM) with bilayer pepsin structure for protein hydrolysis. Sep. Purif. Technol..

[22] Wang J., Tian J., Gao S., Shi W. (2020). Dopamine triggered one step polymerization and codeposition of reactive surfactant on PES membrane surface for antifouling modification. Sep. Purif. Technol..

[23] Muñiz-Mouro A., Gullón B., Lu-Chau T.A., Eibes G. (2020). Green and sustainable synthesis of oligorutin using an enzymatic membrane reactor: Process optimization. Food Bioprod. Process.

[24] Rasouli H., Iliuta I., Bougie F., Garnier A., Iliuta M.C. (2021). Enzyme-immobilized flat-sheet membrane contactor for green carbon capture. Chem. Eng. J..

[25] Zhang H., Luo J., Li S., Wei Y., Wan Y. (2018). Biocatalytic membrane based on polydopamine coating: A platform for studying immobilization mechanisms. Langmuir.

[26] Su Z., Luo J., Sigurdardóttir S.B., Manferrari T., Jankowska K., Pinelo M. (2021). An enzymatic membrane reactor for oligodextran production: Effects of enzyme immobilization strategies on dextranase activity. Carbohyd. Polym..

[27] Zhou F., Luo J., Song S., Wan Y. (2020). Nanostructured polyphenol-mediated coating: A versatile platform for enzyme immobilization and micropollutant removal. Ind. Eng. Chem. Res..

[28] Touqeer T., Mumtaz M.W., Mukhtar H., Irfan A., Akram S., Shabbir A., Rashid U., Nehdi I.A., Choong T.S.Y. (2019). Fe_3_O_4_-PDA-lipase as surface functionalized nano biocatalyst for the production of biodiesel using waste cooking oil as feedstock: Characterization and process optimization. Energies.

[29] Li Y., Shi S., Cao H., Zhao Z., Su C., Wen H. (2018). Improvement of the antifouling performance and stability of an anion exchange membrane by surface modification with graphene oxide (GO) and polydopamine (PDA). J. Membr. Sci..

[30] Zarghami S., Mohammadi T., Sadrzadeh M. (2019). Preparation, characterization and fouling analysis of in-air hydrophilic/underwater oleophobic bio-inspired polydopamine coated PES membranes for oily wastewater treatment. J. Membr. Sci..

[31] Barbosa O., Ortiz C., Berenguer-Murcia A., Torres R., Rodrigues R.C., Fernandez-Lafuente R. (2013). Glutaraldehyde in bio-catalysts design: A useful crosslinker and a versatile tool in enzyme immobilization. RSC Adv..

[32] Neelam V., Lovely S., Anjum G., Vinita H., Vikas H. (2020). Novel approach using activated cellulose film for efficient immobilization of purified diamine oxidase to enhance enzyme performance and stability. Prep. Biochem. Biotech..

[33] Xu J., Du P., Bi W., Yao G., Li S., Liu H. (2019). Graphene oxide aerogels co-functionalized with polydopamine and polyethylenimine for the adsorption of anionic dyes and organic solvents. Chem. Eng. Res. Des.

[34] Kondabagil K., Karanth N.G., Sajja H.K., Divakar S. (2000). An esterification method for determination of lipase activity. Biotechnol. Lett..

[35] Wang F., Luo X., Guo J., Zhang W. (2021). Treatment of soy sauce wastewater with biomimetic dynamic membrane for colority removal and chemical oxygen demand lowering. An. Acad. Bras. Cienc..

[36] Ardhaoui M., Falcimaigne A., Engasser J.-M., Moussou P., Pauly G., Ghoul M. (2004). Acylation of natural flavonoids using lipase of candida antarctica as biocatalyst. J. Mol. Catal B Enzym..

[37] Geng X., Wang J., Ye J., Yang S., Han Q., Lin H., Liu F. (2020). Electrosprayed polydopamine membrane: Surface morphology, chemical stability and separation performance study. Sep. Purif. Technol..

[38] Zhu L., Jiang J., Zhu B., Xu Y. (2011). Immobilization of bovine serum albumin onto porous polyethylene membranes using strongly attached polydopamine as a spacer. Colloids Surf. B Biointerfaces.

[39] Elias N., Wahab R.A., Jye L.W., Mahat N.A., Chandren S., Jamalis J. (2021). Taguchi orthogonal design assisted immobilization of candida rugosa lipase onto nanocellulose-silica reinforced polyethersulfone membrane: Physicochemical characterization and operational stability. Cellulose.

[40] Huang X., Ge D., Xu Z. (2007). Preparation and characterization of stable chitosan nanofibrous membrane for lipase immobilization. Eur. Polym. J..

[41] Ye P., Jiang J., Xu Z. (2007). Adsorption and activity of lipase from candida rugosa on the chitosan-modified poly(acrylonitrile-co-maleic acid) membrane surface. Colloids Surf. B Biointerfaces.

[42] Asmat S., Anwer A.H., Husain Q. (2019). Immobilization of lipase onto novel constructed polydopamine grafted multiwalled carbon nanotube impregnated with magnetic cobalt and its application in synthesis of fruit flavours. Int. J. Biol. Macromol..

[43] Chao C., Liu J., Wang J., Zhang Y., Zhang B., Zhang Y., Xiang X., Chen R. (2013). Surface modification of halloysite nanotubes with dopamine for enzyme immobilization. ACS Appl. Mater. Interfaces.

[44] Song J., He W., Shen H., Zhou Z., Li M., Su P., Yang Y. (2019). Construction of multiple enzyme metal–organic frameworks biocatalyst via DNA scaffold: A promising strategy for enzyme encapsulation. Chem. Eng. J..

[45] Luo J., Meyer A.S., Mateiu R.V., Kalyani D., Pinelo M. (2014). Functionalization of a membrane sublayer using reverse filtration of enzymes and dopamine coating. ACS Appl. Mater. Interfaces.

[46] Emese B., Daniel B., Anamaria T., Péter F., Szilvia K., Tivadar F. (2016). Recyclable solid-phase biocatalyst with improved stability by sol-gel entrapment of beta-D-galactosidase. J. Mol. Catal B. Enzym..

[47] Temkov M., Petrovski A., Gjorgieva E., Popovski E., Lazarova M., Boev I., Paunovic P. (2019). Inulinase immobilization on polyethylene glycol/polypyrrole multiwall carbon nanotubes producing a catalyst with enhanced thermal and operational stability. Eng. Life Sci..

[48] Luo Q., Huang Q., Chen Z., Yao L. (2018). Temperature dependence of the pore structure in polyvinylidene fluoride (PVDF)/graphene composite membrane probed by electrochemical impedance spectroscopy. Polymers.

[49] Wang H., Jia C., Xia X., Karangwa E., Zhang X. (2017). Enzymatic synthesis of phytosteryl lipoate and its antioxidant properties. Food Chem..

